# Natural Polymers in Guided Bone Regeneration (GBR)

**DOI:** 10.3390/jfb17070331

**Published:** 2026-07-07

**Authors:** Anca Fratila, Diana Marian, Alexandru Petre, Anca Hermenean, Ioana Lile

**Affiliations:** 1 Department of Dental Medicine and Nursing, Faculty of Medicine, “Lucian Blaga” University of Sibiu, 550169 Sibiu, Romania; anca.fratila@ulbsibiu.ro; 2 Military Clinical Emergency Hospital of Sibiu, 550024 Sibiu, Romania; 3 Department of Dentistry, Faculty of Dentistry, “Vasile Goldiș” Western University of Arad, 94–96 Revolutiei Boulevard, 310025 Arad, Romania; 4 Department of Prosthodontics, “Carol Davila” University of Medicine and Pharmacy, 050474 Bucharest, Romania; alexandru.petre@umfcd.ro; 5 “Aurel Ardelean” Institute of Life Sciences, Vasile Goldis Western University of Arad, 310025 Arad, Romania

**Keywords:** guided bone regeneration, collagen, chitosan, alginate, composite materials

## Abstract

Guided Bone Regeneration (GBR) is a pivotal technique in dental and orthopedic applications for regenerating bone in areas of deficiency. Natural polymers such as collagen, chitosan, alginate, and gelatin have emerged as essential materials in GBR due to their biocompatibility, biodegradability, and bioactivity. These polymers not only provide a scaffold for bone regeneration but also support cellular adhesion, proliferation, and differentiation. Despite their benefits, challenges such as variable degradation rates, insufficient mechanical strength, and limited bioactivity hinder their optimal clinical use. To address these limitations, ongoing research focuses on enhancing the properties of natural polymers. Composite materials combining fast- and slow-degrading polymers are being developed to achieve consistent degradation rates. Surface modifications, including nanoscale texturing and growth factor coatings, are improving bioactivity. Nanotechnology further enhances the structural and therapeutic potential of GBR materials, while advancements in 3D bioprinting enable the creation of customized scaffolds with precise architecture. These innovations aim to bridge the gap between biological compatibility and clinical functionality, making natural polymers more adaptable and effective in GBR. This review highlights the mechanisms, challenges, and advancements in natural polymers for GBR, emphasizing their potential to transform bone regeneration into a more reliable and patient-centered approach.

## 1. Introduction

Guided Bone Regeneration (GBR) is a technique used in dentistry to help regrow bone in areas where it has been lost or damaged. It works by placing a barrier, usually a membrane, over the area that needs bone regeneration to block soft tissue invasion [1]. By creating a protected space just for bone cells to grow, GBR makes it possible to rebuild bone even in challenging spots. This technique has become essential in dental work, being among the most common techniques for reconstructing alveolar bone and addressing peri-implant bone deficiencies [2].

GBR plays a vital role in modern dental treatments because it helps create a solid foundation for implants and other restorative procedures. Many people suffer from bone loss due to factors like gum disease, injury, or extended tooth loss, which can leave them with insufficient bone to support implants [3]. Guided bone regeneration (GBR) helps rebuild bone to provide stable support for implants, enhancing their function, appearance, and longevity. It reduces implant failure and supports long-term success [4].

Natural polymers, like collagen, chitosan, and alginate, have become important materials in GBR because they are compatible with the body and support the bone-healing process. These polymers are used to make GBR membranes that act as scaffolds, giving bone cells a safe place to grow while keeping unwanted tissue out. Because natural polymers are similar to the body’s own materials, they are less likely to cause a reaction, which makes the healing process smoother. Many of these materials are also biodegradable, meaning they gradually dissolve over time and do not require a second surgery to remove them. Some natural polymers even encourage bone growth through their bioactive properties, helping to support natural bone healing. These properties make natural polymers attractive candidates for GBR applications; however, their clinical effectiveness remains dependent on material-specific characteristics and clinical conditions.

## 2. Principles of Guided Bone Regeneration (GBR)

**Figure 1:**
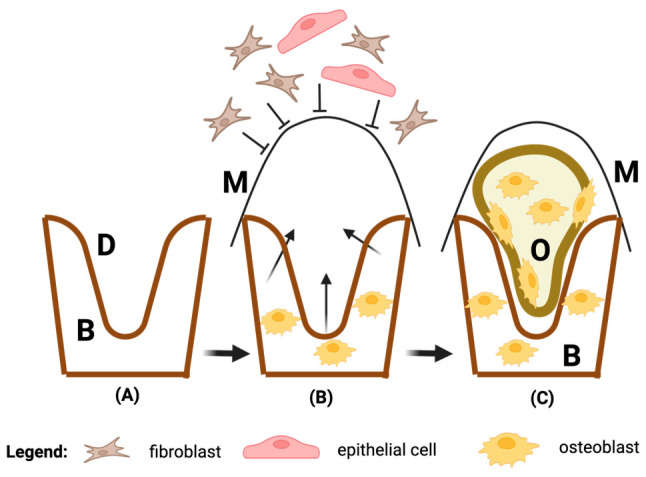
Guided bone regeneration (GBR) mechanism. (**A**) Bone defect. (**B**) The membrane barrier prevents the penetration of epithelial cells and fibroblasts and allows access to the defect of osteoprogenitor cells originating from the native bone. (**C**) Regeneration of the bone defect. Abbreviations: B—bone; D—defect; M—membrane barrier; O—osteoid (non-mineralized bone); Created in BioRender. Hermenean, A. (2026). https://BioRender.com/c75z741.

Insufficient horizontal or vertical bone at implant sites can cause clinical challenges and must be corrected before implant placement, often through ridge augmentation. Guided Bone Regeneration (GBR) is a common ridge augmentation technique using barrier membranes, sometimes with bone grafts or substitutes. GBR promotes bone growth by allowing osteogenic cells (e.g., osteoblasts derived from the periosteum or adjacent bone or bone marrow) to reach the defect site while blocking cells that hinder bone formation. For effective GBR, four principles are essential: blocking epithelial and connective tissue, maintaining space, stabilizing the fibrin clot, and achieving primary wound closure. Following GBR, bone regeneration progresses in stages (Figure 1). Within 24 h, a blood clot forms, releasing growth factors that attract immune cells. The clot is replaced by granulation tissue, rich in blood vessels that transport nutrients and stem cells for osteoid formation. This develops into woven bone, which matures into lamellar bone over 3–4 months, forming stable bone with mature marrow [4].

## 3. Natural Polymers vs. Synthetic Polymers in GBR

Natural polymers are materials obtained from biological sources, including plants, animals, and microorganisms. In guided bone regeneration (GBR), their use has expanded considerably due to their high compatibility with the human body and their capacity to promote tissue repair. Common examples in dentistry include collagen, chitosan, alginate, and gelatin. These substances are valued because they closely mimic the architecture of natural tissues, which facilitates their interaction with human cells. In contrast to synthetic materials, many natural polymers exhibit inherent bioactivity, meaning they can actively support essential cellular functions such as adhesion, proliferation, and differentiation—key processes for effective bone regeneration.

One of the main benefits of natural polymers is their excellent biocompatibility, which minimizes the likelihood of inflammatory reactions or immune rejection. Another important advantage is their biodegradability, as many of these materials are gradually broken down within the body. Additionally, they can stimulate a favorable biological response, creating optimal conditions for bone healing. Because they degrade over time, they often eliminate the need for a second surgical procedure to remove the membrane, making the overall treatment less invasive and more comfortable for patients.

To function effectively in GBR applications, natural polymers must meet several essential criteria. First, they need to be biocompatible, ensuring that they do not trigger adverse reactions that could interfere with healing. Materials such as collagen and chitosan are widely used for this reason. Controlled degradability is another critical requirement; the material should persist long enough to support the initial healing phase and then gradually resorb as new bone forms.

Mechanical strength is also important, as GBR membranes must act as stable barriers that prevent soft tissue from invading the regeneration site. Therefore, the material must withstand the mechanical forces present in the oral environment without collapsing or tearing prematurely. In addition, the bioactivity of certain natural polymers, particularly collagen, enhances the regenerative process by promoting cellular interactions and tissue formation. Porosity is another key feature, allowing the diffusion of nutrients and oxygen to the healing site while still maintaining an effective barrier against unwanted tissue infiltration.

The performance of GBR membranes is strongly influenced by the anatomical location of the defect and the local biomechanical environment [5]. Defects in areas exposed to significant functional loading, such as posterior mandibular regions, require membranes with greater mechanical stability and prolonged barrier function [6]. In contrast, contained defects with lower mechanical demands may benefit from highly bioactive and rapidly resorbable materials. Therefore, the optimal characteristics of a GBR membrane cannot be generalized across all clinical situations and should be considered in relation to site-specific biomechanical requirements.

When compared with synthetic polymers used in dentistry, each group presents distinct advantages and limitations. Natural polymers, such as collagen and chitosan, offer superior biocompatibility and bioactivity, making them especially suitable for GBR procedures. On the other hand, synthetic polymers like polylactic acid (PLA) and polyglycolic acid (PGA) are known for their high mechanical strength and the ability to be engineered with precise properties, including controlled degradation rates. However, they may lack the same level of biological compatibility and activity, which can sometimes lead to inflammatory responses or require additional modifications to enhance cell attachment and growth. These contrasting findings suggest that no single membrane currently fulfills all GBR requirements, highlighting the need for hybrid or composite systems capable of combining biological performance with mechanical reliability.

Table 1 provides a comparative overview of the strengths and limitations of natural and synthetic polymers for GBR applications in dentistry, based on current research findings.

In dentistry, the choice between natural and synthetic polymers often depends on the specific needs of the patient and the type of procedure. For example, in cases where high mechanical stability is essential, synthetic polymers may be favored. However, for patients who would benefit from a more biologically interactive and resorbable material, natural polymers are often the preferred choice. Overall, natural polymers offer several biological advantages for GBR applications, particularly regarding biocompatibility and tissue integration, although their performance may be limited by mechanical and degradation-related factors.

## 4. Types of Natural Polymers Used in GBR

### 4.1. Collagen: Structure, Properties, and Uses in GBR

Collagen is one of the most used natural polymers in guided bone regeneration due to its structural similarity to the body’s own tissues. As a protein that naturally occurs in skin, bones, tendons, and other connective tissues, collagen provides a biocompatible framework that supports cell attachment and tissue growth. Essential in the extracellular matrix (ECM), collagen supports tissue formation and imparts mechanical and biochemical properties. Structurally, collagen is composed of long chains of amino acids that form a triple-helix [19], formed by repeating Gly-X-Y amino acid sequences, where glycine is every third residue, often with proline and hydroxyproline [20]. This helical structure, primarily found in types I, II, and III collagen, is stabilized by hydrogen bonds and cross-linked by both enzymatic and non-enzymatic processes. Collagen molecules self-assemble into fibrils with a characteristic staggered pattern, stabilized by cross-links, crucial for mature collagen’s stability and function in tissues [21,22].

These qualities make collagen an ideal scaffold in GBR, as it can act as a barrier that supports new bone formation while maintaining stability against forces in the oral environment. In GBR, collagen membranes are widely used due to their ability to protect the regenerative area from soft tissue invasion, allowing bone cells to grow without interference. Collagen is also biodegradable, meaning it gradually degrades as the bone heals, often eliminating the need for a second procedure to remove the membrane. Additionally, collagen’s natural bioactivity encourages cellular attachment and signaling, which can further stimulate bone healing and regeneration.

Collagen-based membranes remain the most widely used biomaterials in clinical GBR procedures and represent the current benchmark for regenerative membrane performance. Several commercially available products, including Bio-Gide^®^, Jason^®^ Membrane, and Ossix^®^ membranes, have demonstrated favorable clinical outcomes due to their excellent biocompatibility, tissue integration, and ability to support bone regeneration [23]. However, these materials may exhibit limitations related to mechanical stability, space maintenance in larger defects, and variability in degradation rates depending on membrane composition and cross-linking methods. Such limitations have stimulated the development of alternative natural polymer-based systems, including chitosan-, alginate-, gelatin-, and cellulose-derived materials, which are being investigated for their potential to provide improved mechanical performance, controlled degradation, antimicrobial activity, and enhanced delivery of bioactive molecules [24]. While these novel materials show promising preclinical results, collagen-based membranes remain the most extensively validated natural biomaterials in current clinical practice.

### 4.2. Chitosan: Biocompatibility and Barrier Role in GBR

Chitosan is a natural polymer derived from chitin, a substance found in the shells of crustaceans like shrimp and crabs. Known for its excellent biocompatibility and mild antimicrobial properties, chitosan has become popular in GBR applications. As a barrier material, chitosan effectively prevents soft tissue from entering the bone regeneration site, creating a protected space for bone cells to proliferate. Chitosan’s antimicrobial properties also reduce the risk of infection, an important factor in oral surgeries and regenerative procedures.

In addition to being biocompatible, chitosan is also biodegradable, gradually breaking down as bone regeneration progresses. This means chitosan membranes provide essential support early in the healing process and then naturally dissolve, allowing the newly formed bone to integrate seamlessly into the surrounding tissue. Furthermore, chitosan’s structure can be modified to enhance its mechanical properties, making it suitable for use in various GBR applications where both stability and controlled degradation are needed (Table 2).

### 4.3. Alginate: Flexibility and Use in GBR Matrices

Alginate is a naturally occurring polymer derived from brown seaweed and is valued in GBR for its flexibility and adaptability. Made up of blocks of mannuronic and guluronic acid, alginate can form a gel-like structure that is flexible yet strong, making it an ideal material for creating matrices in GBR. Alginate’s gel-forming ability enables it to adapt to the contours of the bone defect, providing a custom-fit scaffold for bone regeneration.

One of the main advantages of alginate is its ease of modification; it can be combined with other materials, such as hydroxyapatite, to create composite matrices with enhanced properties [41]. Alginate-based gels are also highly porous, allowing the flow of nutrients and oxygen, which supports cellular activity and bone growth [42]. This flexibility and adaptability make alginate an appealing choice for GBR, particularly in cases requiring a more dynamic, form-fitting scaffold that can still degrade naturally as healing occurs (Table 3).

### 4.4. Gelatin: Resorbability and Applicability in GBR

Gelatin is a natural polymer derived from collagen, and it shares many of collagen’s beneficial properties, including biocompatibility and biodegradability (Table 4). In GBR, gelatin’s main advantage is its rapid resorbability, as it breaks down faster than some other natural polymers, making it ideal for cases where short-term support is needed. Gelatin can also be modified to control its degradation rate, allowing it to adapt to the specific requirements of the GBR procedure.

Due to its resorbability, gelatin is often used in combination with other materials to create composite membranes that provide initial support for bone growth while gradually dissolving to integrate with the newly formed tissue (Table 4). Additionally, gelatin is easy to process and can form porous scaffolds, which allow for better cell infiltration and nutrient flow. Its versatility and ease of use make gelatin a valuable polymer in GBR, particularly when used as a temporary matrix that fosters rapid healing and resorption.

### 4.5. Cellulose Derivatives: Mechanical Stability and Application in GBR

Cellulose, a natural polymer found in plant cell walls, can be processed into various derivatives—such as methylcellulose and hydroxyethyl cellulose—that offer enhanced properties for GBR applications [60]. Cellulose derivatives are known for their excellent mechanical stability, which makes them strong and resilient enough to act as barriers in the GBR process (Table 5). This mechanical strength is particularly valuable in areas where the membrane needs to withstand physical pressures without collapsing.

In addition to stability, cellulose derivatives are biocompatible and can be modified to control their degradation rates, making them suitable for longer-term support in GBR. Cellulose membranes can be structured to have different porosities, allowing nutrient flow while still preventing unwanted soft tissue invasion. Their ability to provide durable support while being biocompatible and resorbable makes cellulose derivatives a versatile choice for GBR, particularly in situations where structural integrity is a priority.

### 4.6. Hyaluronic Acid: Properties and Applications in GBR

Hyaluronic acid (HA) is a naturally occurring glycosaminoglycan widely distributed throughout the extracellular matrix of connective tissues. Due to its excellent biocompatibility, biodegradability, high water-retention capacity, and ability to regulate cellular migration and proliferation, HA has attracted considerable interest in tissue engineering and regenerative medicine [69]. In GBR applications, hyaluronic acid is most commonly incorporated into hydrogels, composite scaffolds, and bioactive delivery systems rather than being used as a stand-alone barrier membrane [70].

HA-based biomaterials contribute to tissue regeneration by providing a hydrated microenvironment that supports cell infiltration, angiogenesis, and extracellular matrix remodeling [71]. In addition, HA can serve as a carrier for growth factors, drugs, and osteogenic molecules, enabling their controlled release at the defect site [72]. Several studies have demonstrated that HA-containing hydrogels and composite scaffolds promote osteoblast activity, enhance bone formation, and improve tissue integration [70]. These properties make hyaluronic acid a valuable component of multifunctional GBR systems, particularly when combined with other natural polymers such as collagen, gelatin, or chitosan to improve mechanical stability and regenerative performance.

## 5. Mechanisms of Action of Natural Polymers in GBR

### 5.1. Biocompatibility and Tissue Integration

One of the fundamental reasons for using natural polymers in guided bone regeneration (GBR) is their high biocompatibility. Biocompatibility means that these materials work harmoniously with the body’s tissues without causing adverse reactions, such as inflammation or rejection. Since natural polymers like collagen, chitosan, and alginate are structurally similar to the body’s own biological materials, they are readily accepted by the body’s immune system, minimizing the risk of inflammatory responses that can impede bone healing. Studies have shown that chitosan and its derivatives exhibit excellent biocompatibility and osteoconductivity, enhancing cellular attachment and bone formation while avoiding inflammatory reactions [30,73].

In addition to being biocompatible, many natural polymers support seamless integration with surrounding tissues. For example, collagen, a protein naturally found in bones and connective tissues, encourages bone cells to adhere to and grow on its surface [74]. This compatibility fosters better integration between the polymer and the regenerating bone tissue, creating a cohesive and stable environment that supports the body’s natural healing process. Studies confirm that collagen-based membranes can promote cellular adhesion and bone regeneration through their structural and biochemical compatibility [15,75].

Chitosan, when combined with other natural polymers like alginate, forms composites that are particularly effective for GBR. These composites enhance the biomechanical properties and bioactivity of scaffolds, fostering a conducive environment for osteogenesis [76].

By encouraging cellular attachment and growth, biocompatible natural polymers help establish a stable scaffold for new bone to form and thrive, leading to more effective and predictable GBR outcomes. For example, chitosan–alginate hydrogels [55,77] and collagen–chitosan membranes [75] have demonstrated enhanced osteoinductive and osteoconductive properties, supporting the formation of new bone tissue in vivo.

### 5.2. Mechanical Stability and Soft Tissue Invasion Prevention

In GBR, it is crucial that the barrier membrane remains intact and stable throughout the healing process to effectively guide bone regeneration. Natural polymers used in GBR provide the necessary mechanical stability to withstand the pressures present in the oral cavity, such as those caused by chewing, movement, and other forces. This stability is vital because a durable membrane prevents the collapse or displacement that could disrupt the healing process. Studies show that certain natural polymer-based membranes, such as those incorporating chitosan and collagen, exhibit excellent mechanical properties and stability, which make them well-suited for GBR application [29,77].

Beyond structural stability, natural polymers act as a physical barrier that blocks soft tissue invasion, which is one of the main obstacles in GBR. Without this barrier, soft tissues like gums and connective tissue would rapidly fill the bone defect site, competing with bone cells and preventing bone from regenerating properly. Barrier membranes based on biocompatible polymers like collagen have been shown to provide an effective exclusion of soft tissue, thereby maintaining space for bone formation [78,79].

By acting as a fence around the bone regeneration area, natural polymer membranes keep out unwanted tissue, allowing only bone cells to populate the space. This selective space management ensures that the regenerating bone has an unobstructed environment to grow, leading to a more successful regeneration process. Novel bilayer membranes with enhanced mechanical strength and controlled biodegradation rates further demonstrate the capability to prevent soft tissue invasion while supporting osteogenesis [11,80].

Although natural polymers demonstrate favorable biological properties, their mechanical performance remains highly dependent on the clinical setting. Collagen-based membranes may provide adequate performance in small or contained defects but can exhibit limited space-maintaining capacity in larger defects or mechanically demanding environments [81]. Consequently, many studies have explored cross-linking strategies, composite formulations, or reinforcement with inorganic phases to improve structural stability. However, direct comparisons between different membrane systems under clinically relevant loading conditions remain limited.

### 5.3. Controlled Degradability and Gradual Release of Growth Factors

An ideal natural polymer for GBR not only provides temporary support but also degrades at a controlled rate that matches the pace of bone regeneration. Controlled degradability means that the material gradually dissolves as the new bone tissue takes shape, which eliminates the need for a second surgery to remove the membrane. This gradual degradation ensures that the scaffold is present only as long as it is needed, providing initial stability and then disappearing as the bone regenerates and takes over the area. Research highlights that hydrogel scaffolds incorporating chitosan and collagen can achieve controlled degradation while supporting osteogenesis [82,83].

Additionally, certain natural polymers can be engineered to release growth factors or other bioactive agents as they degrade, which can actively promote bone healing. Growth factors are proteins that signal cells to grow, divide, and specialize—key actions needed for bone regeneration. For instance, collagen and chitosan scaffolds loaded with growth factors, such as bone morphogenetic proteins (BMPs), demonstrate sustained release during degradation. These growth factors stimulate osteogenesis, promoting bone cell proliferation and differentiation [84,85].

By combining controlled degradation with bioactive molecule delivery, natural polymers used in GBR provide a dual benefit: they act as a stable scaffold initially and then stimulate bone growth through growth factor release as they gradually dissolve. For example, BMP-2 loaded hydrogels have shown enhanced bone regeneration while eliminating the need for high doses of growth factors, making this approach more efficient [86].

A persistent controversy concerns the optimal degradation rate of GBR membranes. While rapid degradation may facilitate tissue remodeling and reduce foreign-body persistence, premature membrane resorption can result in loss of barrier function and impaired bone regeneration. In contrast, prolonged membrane persistence may maintain structural support but potentially interfere with tissue remodeling and integration [78]. The lack of standardized methodologies for evaluating degradation behavior across studies makes direct comparison difficult and remains an important knowledge gap in the field.

### 5.4. Natural Hydrogels as Delivery Platforms in GBR

Natural hydrogels are a unique and highly adaptable form of natural polymers widely used in guided bone regeneration (GBR). Composed primarily of water within a cross-linked polymer network, hydrogels have a soft, gel-like structure that mimics the body’s natural extracellular matrix, making them highly compatible with bone and soft tissues. Their flexible, shape-conforming nature allows hydrogels to fill irregular bone defects perfectly, creating an ideal microenvironment for bone cell attachment and proliferation. Hydrogels derived from extracellular matrix (ECM) components, such as hyaluronic acid and gelatin, are particularly effective in mimicking the ECM and supporting cell growth [87,88].

One of the key advantages of hydrogels in GBR is their capacity for controlled drug or growth factor release. Hydrogels can be loaded with therapeutic agents, such as antibiotics, anti-inflammatory drugs, or growth factors like bone morphogenetic proteins (BMPs), which are then released gradually over time as the hydrogel degrades. This controlled release is crucial in GBR, as it ensures a sustained supply of bioactive molecules directly at the regeneration site, supporting both the prevention of infection and the stimulation of bone growth. For instance, BMP-2-loaded hydrogels have demonstrated effective and sustained bone regeneration with enhanced mineralization and new bone formation in both in vitro and in vivo models [89,90].

Hydrogels can also be modified to respond to environmental factors, such as pH or temperature, allowing for further customization in release patterns. For example, thermosensitive hydrogels have been engineered to provide sequential release of growth factors, enhancing angiogenesis and osteogenesis in bone defects [89]. This adaptability makes natural hydrogels an invaluable tool in GBR, offering both structural support and a means to deliver drugs and growth factors in a controlled and effective manner.

### 5.5. Porous Structures and Their Applications in GBR

Porous structures within natural polymers play a vital role in enhancing GBR outcomes. A porous structure provides a network of interconnected spaces that allow for the flow of nutrients, oxygen, and cellular waste, creating an ideal environment for bone cell migration and growth. In GBR, porous membranes or scaffolds facilitate better integration with the surrounding tissue, encouraging natural bone formation by supporting the exchange of essential molecules and promoting cellular communication. Studies highlight that porous hydrogels and scaffolds derived from natural polymers like collagen and hyaluronic acid provide structural environments conducive to osteogenesis [78,91,92].

The porosity of the natural polymer scaffold can be tailored to suit specific requirements. For instance, larger pore sizes may be designed to allow for rapid vascularization, as blood vessels can infiltrate the scaffold more easily, delivering oxygen and nutrients directly to the regenerating bone tissue. Smaller pores, on the other hand, help to retain growth factors or drugs within the scaffold, allowing for a slow and steady release as the material degrades. Research supports that tailored pore sizes significantly enhance vascularization and growth factor retention, promoting better healing outcomes [84,93].

Additionally, porous structures provide a greater surface area, which can improve the attachment of cells and the adsorption of growth factors. This increased surface area enhances cellular adhesion and proliferation, providing a stable environment where bone cells can attach, grow, and eventually replace the scaffold material with new bone tissue. The role of high surface area in boosting osteogenesis has been demonstrated in studies utilizing nanofibrous hydrogel membranes and other porous biomaterials [94].

## 6. Challenges and Future Perspectives

While natural polymers have shown great promise in guided bone regeneration (GBR), several limitations still need to be addressed to fully optimize their use in clinical applications. One significant limitation is the variable degradation rates of natural polymers [95]. For example, polymers like gelatin may degrade too quickly, potentially providing inadequate support throughout the entire bone regeneration process, while others like chitosan might degrade too slowly, interfering with the final stages of bone integration [96,97,98]. This inconsistency in degradation can complicate treatment planning, as clinicians need materials that degrade at a predictable rate to match the bone’s natural healing timeline. Another challenge is the limited mechanical strength of many natural polymers. While materials like collagen and alginate provide excellent biocompatibility, they often lack the rigidity needed for load-bearing areas in the jaw or other parts of the mouth that are subject to constant forces from chewing and movement [99,100]. To compensate, these polymers may require reinforcement through additives or combinations with other materials, which can increase complexity and cost. Additionally, some natural polymers lack sufficient bioactivity on their own [4]. Although polymers like collagen naturally encourage cell attachment and growth, others may require additional bioactive molecules or surface modifications to promote cellular responses that are crucial for effective bone regeneration [101].

Ongoing research is focused on improving both the biocompatibility and stability of natural polymers used in GBR to overcome current limitations. One area of interest is the engineering of composite materials that combine different natural polymers to achieve more balanced properties. For instance, by blending a fast-degrading polymer with a slower-degrading one, researchers aim to create materials with more consistent degradation rates, offering reliable support throughout the bone healing process. Composite scaffolds combining natural and synthetic polymers have demonstrated enhanced mechanical properties and controlled degradation, making them suitable for various GBR applications [102,103].

Another avenue of research is exploring surface modifications and chemical treatments that can enhance the bioactivity of natural polymers. Techniques such as coating polymer scaffolds with growth factors or using nanoscale surface texturing can encourage cell attachment, proliferation, and differentiation, even in polymers that are otherwise less bioactive. Recent studies have shown that surface modifications, such as polydopamine coatings or incorporation of bioactive nanoparticles, significantly improve cellular responses and osteointegration [104,105].

Advancements in 3D bioprinting are also opening new possibilities for creating more structurally complex and customized GBR materials. 3D printing allows researchers to design scaffolds with precise control over pore size, shape, and internal architecture, which can enhance both mechanical stability and nutrient flow to the regenerating bone tissue. Patient-specific scaffolds created using 3D bioprinting have shown great promise in improving GBR outcomes through tailored designs and the integration of bioactive components [106,107,108].

Despite significant progress, several unresolved issues remain. First, most available studies are preclinical and use different animal models, defect sizes, and evaluation protocols, limiting cross-study comparisons. Second, natural polymers exhibit substantial batch-to-batch variability depending on their biological source and extraction process, which may influence membrane performance [78]. Third, direct head-to-head comparisons between collagen-, chitosan-, alginate-, gelatin-, and cellulose-based GBR membranes are scarce [55]. Consequently, current evidence does not clearly establish which natural polymer or composite formulation offers the best balance between biocompatibility, degradation kinetics, mechanical stability, and regenerative efficacy. Addressing these gaps will require standardized testing protocols and a greater number of well-designed randomized controlled clinical trials with long-term follow-up. The current scarcity of high-quality clinical evidence remains a major barrier to translating promising preclinical findings into routine clinical practice.

An additional challenge is the limited understanding of how biomechanical factors influence the regenerative performance of natural polymer-based membranes. Most experimental studies are performed under controlled laboratory conditions that do not fully reproduce the complex loading environment encountered in vivo [109]. As a result, the translation of preclinical findings to clinical practice remains difficult. Future studies should incorporate biomechanical testing and site-specific evaluations to determine how membrane composition, degradation behavior, and mechanical properties interact under different clinical conditions.

Interpretation of the available literature is complicated by the considerable heterogeneity of experimental models used to evaluate natural polymers in GBR. While many studies report favorable outcomes, direct comparison is difficult because evidence originates from different biological systems, including cell cultures, rodent models, rabbit models, canine models, and human clinical investigations [110]. Regenerative responses may vary substantially depending on species-specific healing mechanisms, defect characteristics, and study design. Consequently, positive findings reported in one experimental setting cannot necessarily be generalized to other models or clinical applications. The lack of standardized protocols and direct comparative studies represents an important limitation of the current evidence base.

Emerging research is increasingly focused on the development of multifunctional biomaterials capable of actively modulating the regenerative microenvironment [111]. Immunomodulatory membranes designed to regulate macrophage polarization and inflammatory responses, extracellular vesicle-loaded scaffolds for enhanced cell communication, and smart responsive biomaterials capable of adapting to local biological stimuli represent promising strategies for next-generation GBR applications [112]. In parallel, advances in biofabrication technologies and AI-assisted scaffold design may facilitate the development of patient-specific biomaterials with optimized structural, mechanical, and biological properties [113,114]. Although these approaches remain largely experimental, they highlight important future directions for improving the predictability and clinical performance of GBR therapies.

## 7. Conclusions

The integration of natural polymers in guided bone regeneration (GBR) has significantly improved outcomes in dental and orthopedic applications due to their excellent biocompatibility, biodegradability, and ability to support cellular activity. Despite these advantages, limitations such as variable degradation rates and limited mechanical strength still require optimization.

Among the natural polymers currently used in GBR, collagen remains the most clinically established material due to its excellent biocompatibility, bioactivity, and extensive clinical use. However, emerging evidence suggests that composite systems incorporating collagen, chitosan, alginate, or other bioactive components may offer superior performance by combining favorable biological properties with improved mechanical stability and controlled degradation. Despite these advances, broader clinical adoption will require several important milestones, including standardized manufacturing protocols, improved reproducibility, optimization of degradation kinetics and mechanical properties, direct comparative studies between different biomaterial systems, and a greater number of well-designed randomized controlled clinical trials with long-term follow-up. Addressing these challenges will be essential for translating promising experimental findings into predictable and widely accepted clinical therapies.

Current research focuses on improving these materials through composite design, surface modification, and advanced technologies like nanotechnology and 3D bioprinting. These approaches aim to enhance performance and allow better adaptation to individual clinical needs.

Overall, natural polymers remain a promising solution for GBR, with future developments expected to deliver more efficient, personalized, and clinically reliable treatments.

## Figures and Tables

**Table 1:** Comparative Analysis of Natural vs. Synthetic Polymers in Guided Bone Regeneration (GBR).

Type	Advantages	Disadvantages
**Natural Polymers**	High biocompatibility and bioactivity, promoting cellular adhesion and bone integration [7,8]	Limited mechanical strength and faster degradation rates, which may not provide sufficient structural support [9]
	Stronger biofunctionality, with potential for functionalization with bioactive molecules, enhancing osteoconductivity [10]	Variability in degradation rates which may cause premature loss of barrier function needed for GBR [11]
	Enhanced cellular bioactivity, reducing the need for additional growth factors [12]	Limited antimicrobial properties, increasing infection risk without modification [13]
**Synthetic Polymers**	Superior mechanical strength, providing robust support for larger bone defects [14]	Potential cytotoxicity or inflammatory responses in some formulations without bioactive modifications [15]
	Controlled degradation rates, supporting long-term structural integrity in GBR [16]	Requires careful design to avoid overly slow degradation, which may hinder new bone growth [14]
	High adaptability for incorporating antimicrobial agents, reducing infection risk [17]	Reduced osteoconductivity compared to natural polymers, requiring additional bioactive fillers [18]

**Table 2:** Applications of Chitosan-Based Materials in Guided Bone Regeneration.

Materials Used	Key Features	Method of GBR Evaluation	Results	References
Chitosan and derivatives	Evaluation of biocompatibility and efficacy	In vitro studies and osteoconductivity tests	Good osteoconductivity and improved bone regeneration	[25]
Chitosan with nanoparticles	Improving osteoconductivity	In vivo studies on animal models	Accelerated bone regeneration and effective tissue integration	[26]
Asymmetric collagen/chitosan membrane with chitosan nanoparticles loaded with minocycline	Antibacterial and osteogenic properties	In vitro release tests and in vivo studies on animal models	Reduced infections and enhanced osteogenesis	[27]
Chitosan combined with biodegradable polymers	Study on controlled drug release	Controlled release testing and in vitro osteogenicity	Efficient drug release and enhanced osteogenesis	[28]
Chitosan with silver nanoparticles	Antibacterial activity and biocompatibility	In vitro biocompatibility and antibacterial activity tests	Significant reduction of pathogenic bacteria and excellent biocompatibility	[29]
Chitosan with hydroxyapatite	Promoting bone regeneration	Histological evaluation in vivo on animal models	High-quality new bone formation and good implant integration	[30]
Nanofibrous membranes of polycaprolactone/chitosan loaded with metformin	Characterization of fibers, drug release, and in vitro osteogenic activity	In vitro osteogenicity and degradation tests	Stimulates osteoblastic formation and has an adequate degradation rate	[31]
Chitosan with bioactive compounds	Evaluation of osteoinductive properties	Biocompatibility and osteogenic activity studies	Increased osteogenic activity and effective bone integration	[32]
Bioaerogel composite of nano-hydroxyapatite/chitosan	Promoting periodontal regeneration	In vitro and in vivo studies on bone regeneration	Rapid bone formation and excellent periodontal integration	[33]
Chitosan modified with glycerol	Enhancing mechanical strength and biocompatibility	Mechanical and biological studies	Excellent biocompatibility and increased resistance to degradation	[34]
Chitosan with bioapatite nanoparticles	Controlled release of bioactive substances	In vitro and in vivo studies	Good osteoconductivity and bone regeneration	[35]
Chitosan combined with gelatin	Promoting osteogenesis and angiogenesis	Histological tests in vivo	Effective dual stimulation of osteogenesis and angiogenesis	[36]
Chitosan with zinc oxide nanoparticles	Antibacterial and regenerative activity	Antimicrobial and biological studies	Infection reduction and regeneration promotion	[37]
Chitosan with plant-based compounds	Study on natural osteogenicity	In vitro biological tests	Efficient bone regeneration through natural compounds	[38]
Chitosan composite with calcium phosphate	Improving mechanical and regenerative properties	In vitro and in vivo studies	Accelerated osteogenesis and bone integration	[39]
Nanofibrous membranes of chitosan	Excellent biodegradability and mechanical strength	Mechanical and in vivo studies	Controlled biodegradability and good osteoconductivity	[12]
Chitosan with gold nanoparticles	Enhanced regenerative activity	In vitro and in vivo studies	Improved osteogenesis and antibacterial activity	[40]

**Table 3:** Applications of Alginate-Based Materials in Guided Bone Regeneration.

Materials Used	Key Features	Method of GBR Evaluation	Results	References
Alginate composite	Biocompatibility, controlled degradation	Histological and radiological analysis	Enhanced bone regeneration	[43]
Alginate hydrogels	Incorporation of bioactive ions	Micro-CT and mechanical testing	Improved structural integrity and bone growth	[44]
Alginate with ceramic fillers	Enhanced osteoconductivity	Animal model studies	Accelerated osseointegration	[45]
Alginate-based scaffolds	Biofunctionalization with growth factors	In vivo animal tests	Significant increase in bone volume	[46]
Alginate–graphene oxide composites	Improved mechanical properties and bioactivity	Cell culture assays and imaging	Enhanced cellular adhesion and proliferation	[47]
Alginate–chitosan blends	Dual functionality for bone regeneration	Scanning electron microscopy	Enhanced biomineralization	[48]
Alginate scaffolds with protein additives	Controlled release of growth factors	Histological evaluation	Accelerated bone repair	[49]
Injectable alginate solutions	Easy delivery to complex defect sites	Clinical trials	Positive clinical outcomes	[50]
Alginate films	Surface modification for cell attachment	Cell culture experiments	Improved cell differentiation	[51]

**Table 4:** Applications of Gelatin-Based Materials in Guided Bone Regeneration.

Materials Used	Key Features	Method of GBR Evaluation	Results	References
Gelatin scaffolds with bioactive fillers	High porosity, excellent cell adhesion	In vivo animal model	Enhanced bone regeneration and vascularization	[52]
Gelatin with nanoparticle additives	Improved mechanical strength and drug delivery	Micro-CT and histological analysis	Faster defect healing and bone growth	[53]
Gelatin-based hydrogels	Biodegradability and bioactivity	Clinical and laboratory testing	Effective bone defect repair	[54]
Gelatin composites with HA (hydroxyapatite)	Enhanced osteoconductivity and biomineralization	SEM and mechanical tests	Significant increase in bone density	[55]
Gelatin–collagen blends	Mimics natural extracellular matrix	Cell culture and in vivo analysis	Improved osteogenic differentiation	[56]
Gelatin films enriched with growth factors	Sustained release of osteoinductive agents	Bone defect animal models	Accelerated bone formation	[57]
Injectable gelatin-based biomaterials	Easy delivery to complex defect sites	Clinical case studies	Positive outcomes in GBR procedures	[58]
Gelatin–polymer composites	Enhanced mechanical and biological properties	Laboratory and preclinical studies	Better cell proliferation and bone healing	[59]

**Table 5:** Applications of Cellulose -Based Materials in Guided Bone Regeneration.

Materials Used	Key Features	Method of GBR Evaluation	Results	References
Cellulose films	Surface modification for cell attachment	Cell culture experiments	Better cell adhesion and proliferation	[61]
Cellulose nanocrystals	High mechanical strength, excellent bioactivity	In vitro and in vivo studies	Enhanced osteogenic differentiation	[62]
Cellulose composites with bioactive fillers	Enhanced osteoconductivity and drug delivery	SEM and mechanical testing	Significant improvement in bone density	[63]
Nanocellulose hydrogels	Mimics extracellular matrix, high porosity	Animal model studies	Promoted vascularization and bone growth	[64]
Bacterial cellulose scaffolds	Excellent structural stability and biocompatibility	Histological and radiological analysis	Accelerated bone defect healing	[65]
Cellulose membranes enriched with growth factors	Sustained release and osteoinductive properties	In vitro and in vivo evaluation	Enhanced bone volume and regeneration	[66]
Cellulose composites with collagen	Improved mechanical properties and bioactivity	Animal and preclinical studies	Superior defect healing and osseointegration	[67]
Cellulose membranes with functional coatings	Antibacterial properties and high bioactivity	Clinical and laboratory tests	Effective protection and bone growth	[68]

## Data Availability

No new data were created or analyzed in this study. Data sharing is not applicable to this article.
